# Supplementary material for: Pseudoexfoliation syndrome-associated genetic variants affect transcription factor binding and alternative splicing of LOXL1
Source: Nat Commun. 2017 May 23;8:15466. doi: 10.1038/ncomms15466 (PMC5457519; doi:10.1038/ncomms15466)
Supplement: Supplementary Information — Supplementary Figures and Supplementary Tables [file ncomms15466-s1.pdf]

Supplementary Figure 1

Regional association plot in Italian dataset

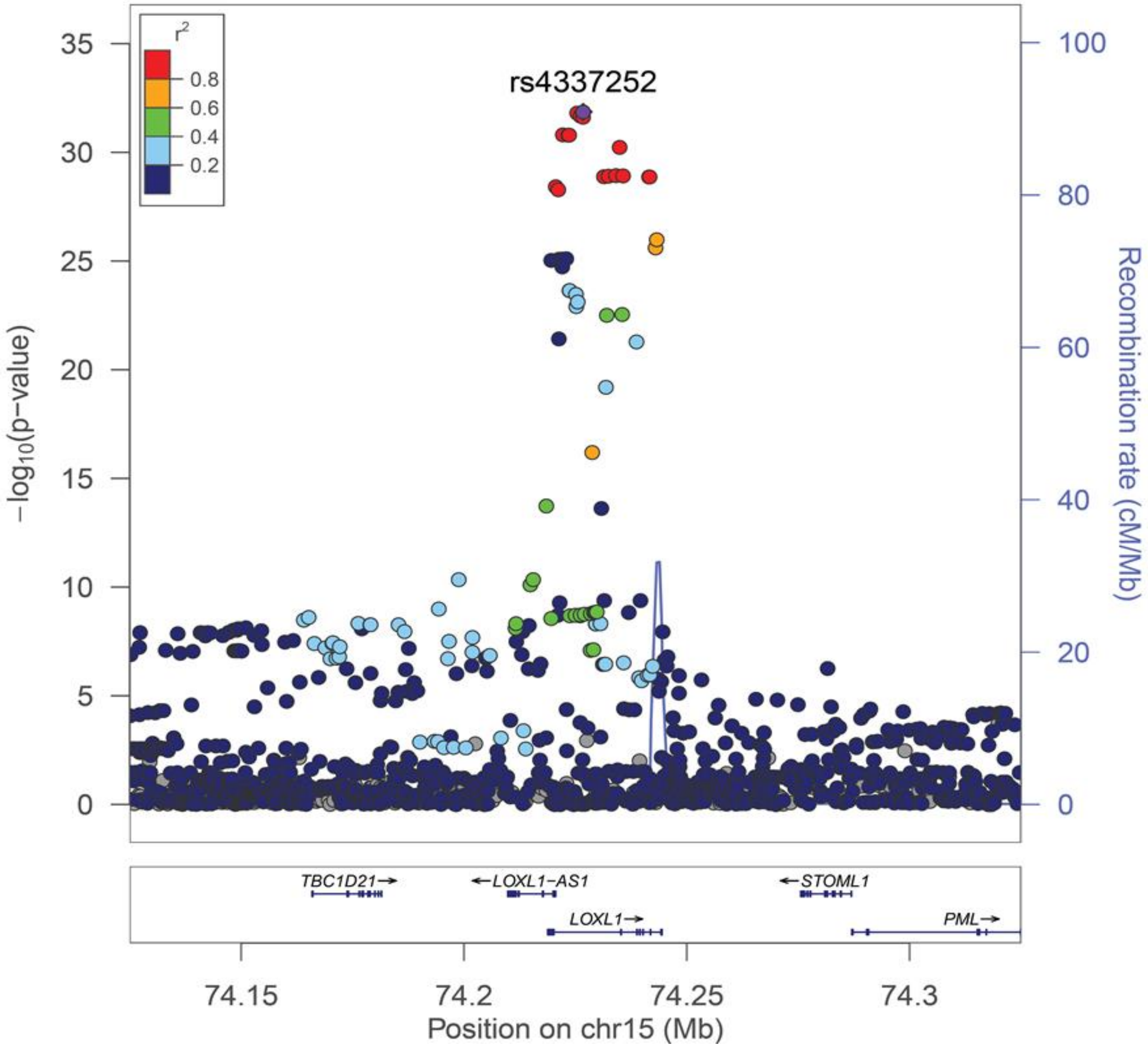

Association results of Italian PEX patients and control cohorts. Regional association plot for *LOXL1* gene with 100 kb upstream and downstream regions: the data of all SNPs are indicated as circles. Association analysis was performed using a logistic regression model adjusted for age and gender. The red and orange circles represent the 14 SNPs showing major association. The left Y-axis represents $-\log_{10}$ p-values and the right Y-axis represents the recombination rate. X-axis represents position of SNPs on chromosome 15 (human genome build GRCh37/Hg19).

Supplementary Figure 2

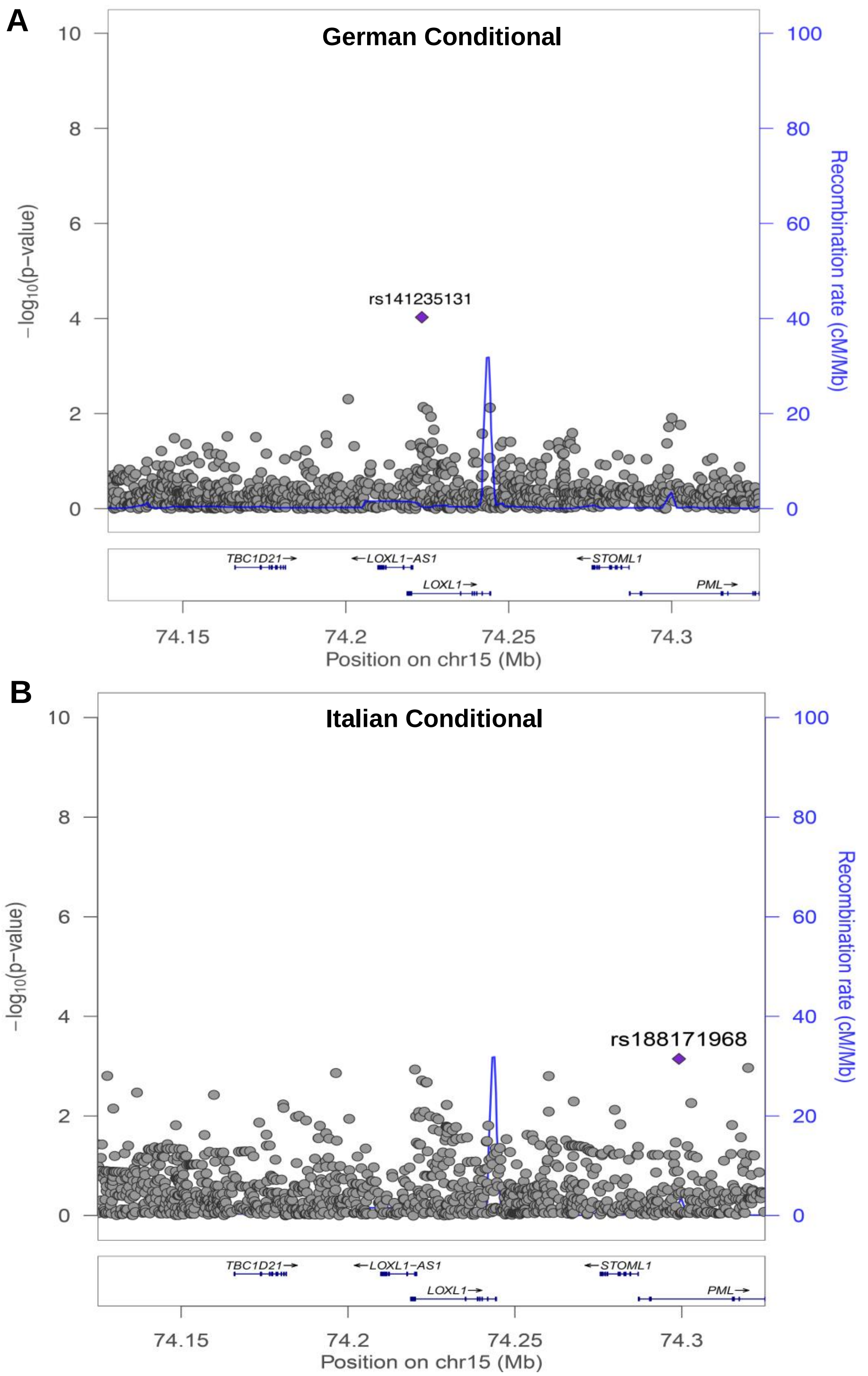

Regional association plot of all SNPs located around the *LOXL1* locus showing the results of conditional analysis in the German (**A**) and Italian (**B**) cohorts on the two coding SNPs rs1048661 and rs3825942.

Supplementary Figure 3

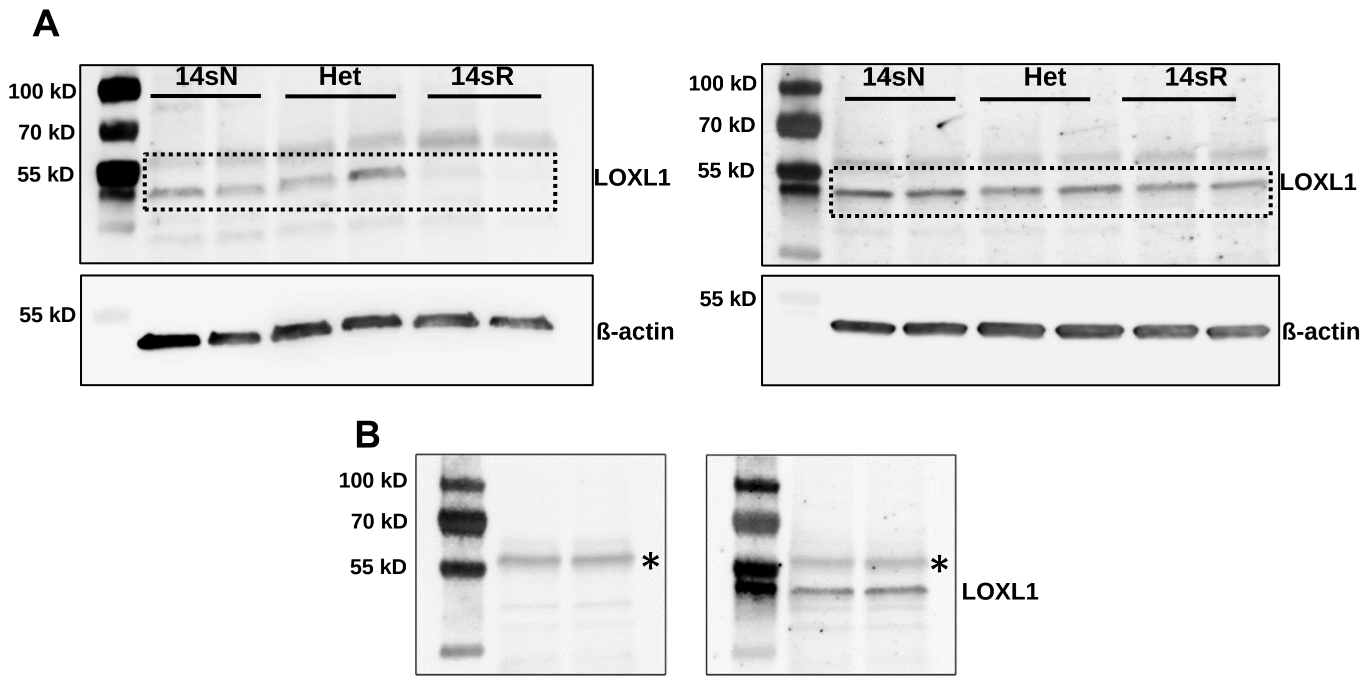

A. Two independent Western blots showing LOXL1 protein expression in iris specimens according to *LOXL1* genotypes; equal loading of samples was verified by immunodetection of ß-actin. A specific band indicating LOXL1 is seen at 52 kDa (14sN, 14 SNP non-risk haplotype in homozygous state; 14sR, 14 SNP risk haplotype in homozygous state; Het, heterozygous allele combinations). **B.** Control blot without (left) and with (right) primary antibody confirming specificity of a LOXL1 band at 52 kD and a non-specific band at 60 kD (asterisk).

C

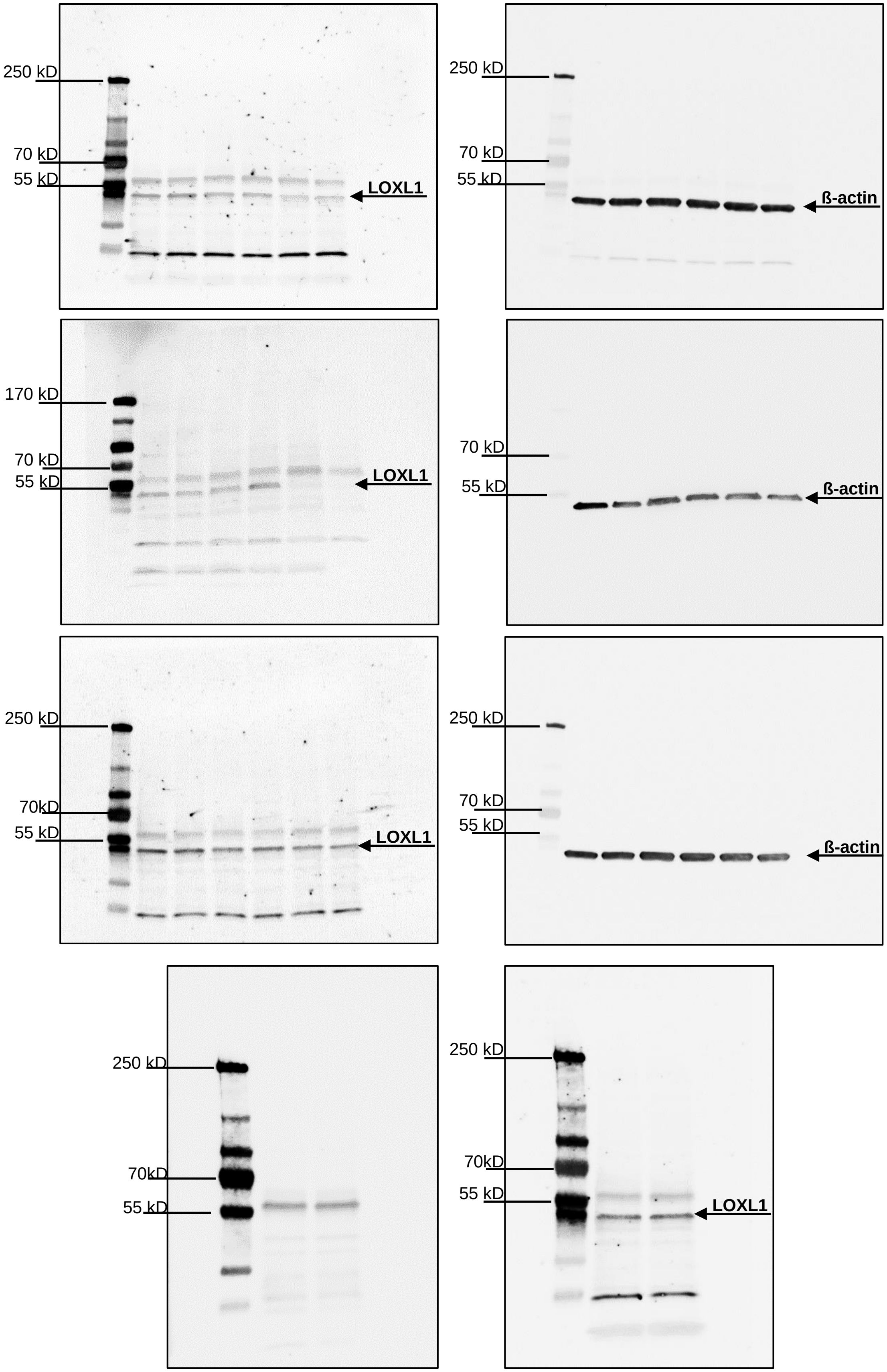

C. Uncropped version of all Western blots shown in Figure 3E and Supplementary Figure 3.

Supplementary Figure 4

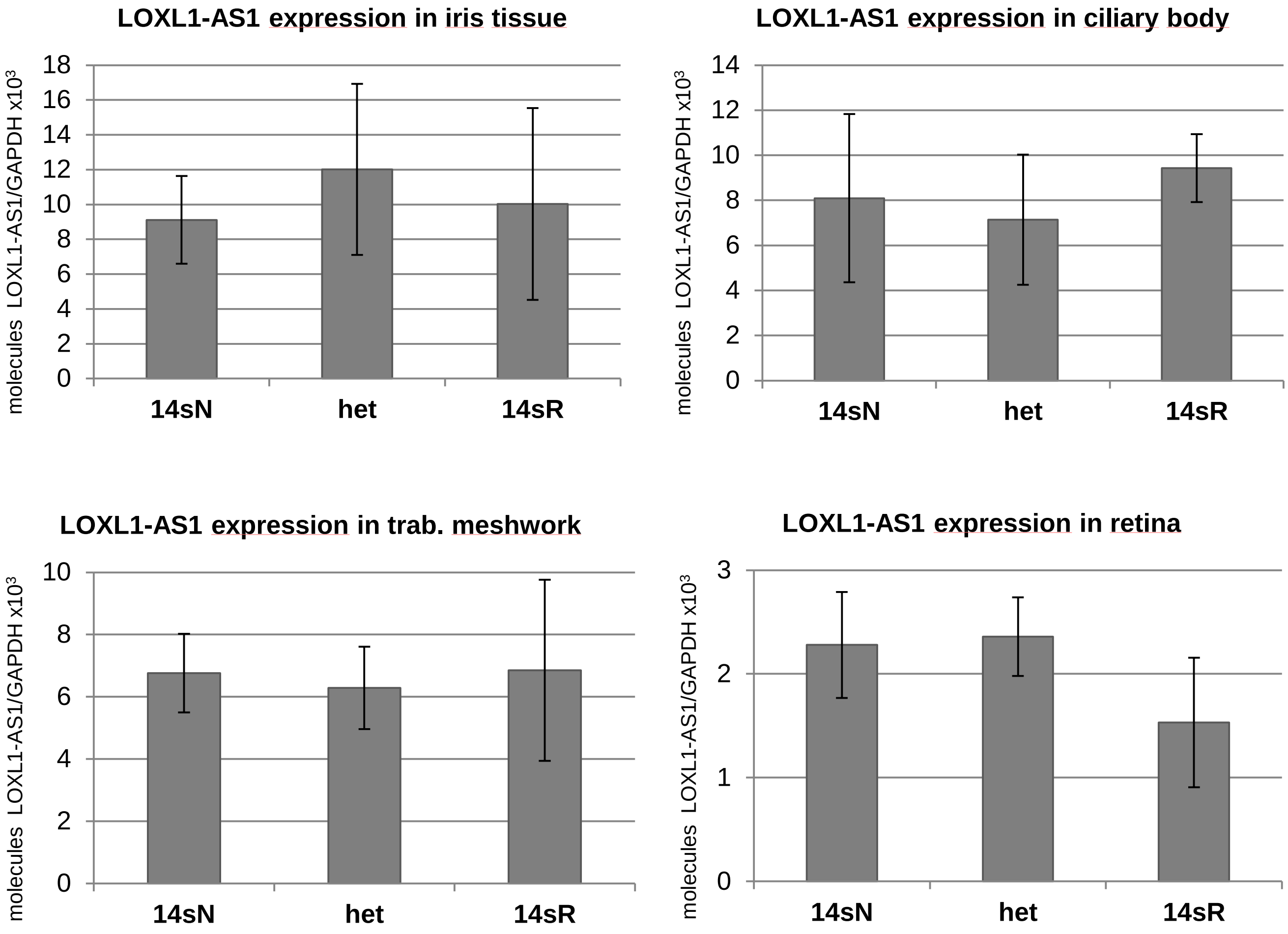

Genotype-correlated expression levels of *LOXL1-AS1* mRNA in iris (n=16), ciliary body (n=16), trabecular meshwork (n=15), and retina tissue samples (n=18) obtained from PEX and control patients using real time PCR technology; data are presented as mean values ± SD (14sN, 14SNP non-risk haplotype in homozygous state; 14sR, 14 SNP risk haplotype in homozygous state; Het, heterozygous allele combinations).

Supplementary Figure 5

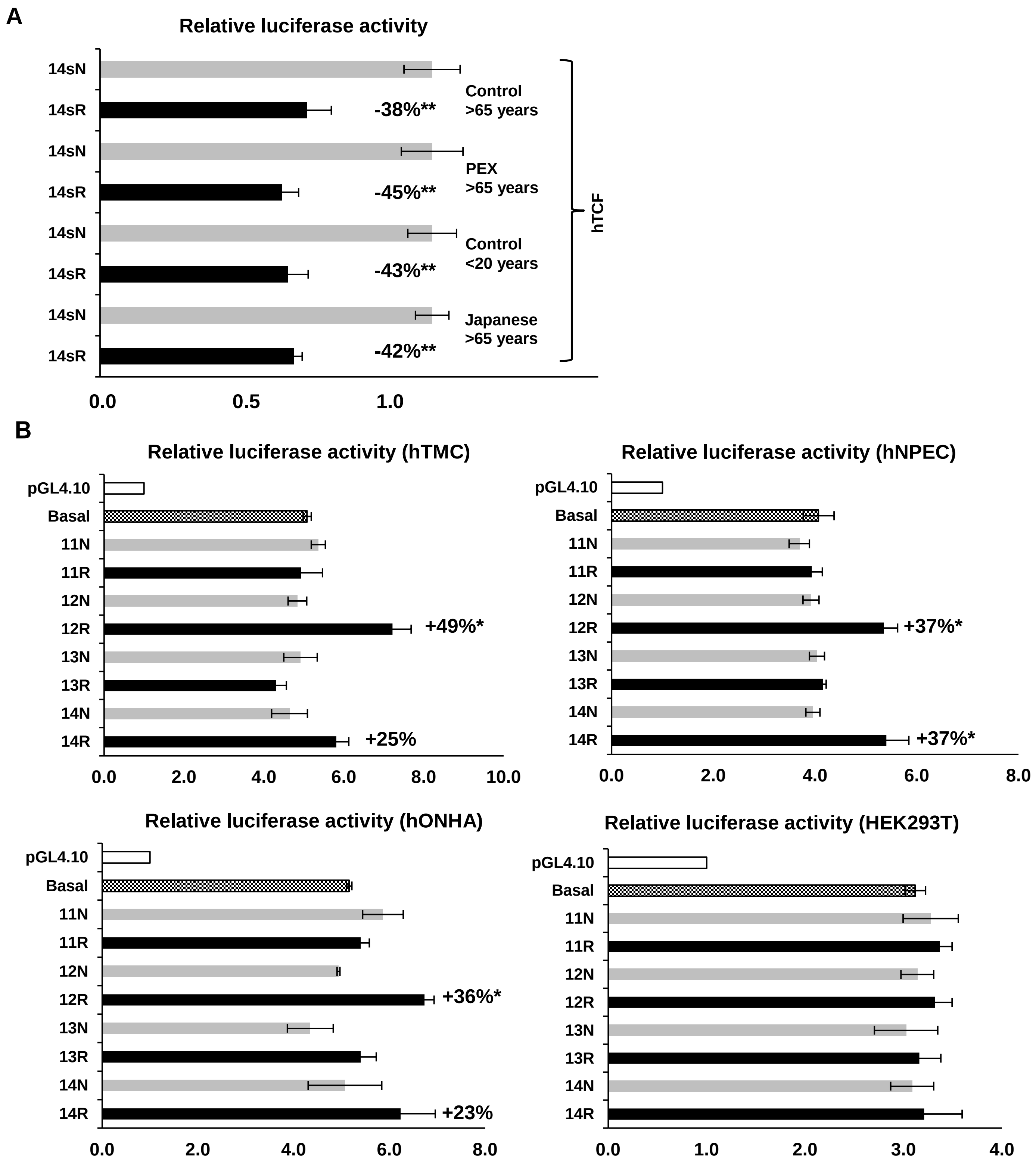

A. Regulatory activity of 14sR (risk) and 14sN (non-risk) sequences in human Tenon's capsule fibroblasts (hTCF) obtained from normal German subjects at older age (control, >65 years) or at younger age (control, <20 years), patients with PEX syndrome (PEX, >65 years), and normal Japanese patients (Japanese, >65 years). Results are expressed as the ratio of Firefly luciferase to Renilla luciferase; the transcriptional activity of the non-risk sequence 14sN was set at 100% (Data represent mean values ± SD of 5 independent experiments; **p<0.005; unpaired two-tailed Student's t-test). **B**. Activity assays using reporter plasmids containing each of the 4 individual risk or non-risk alleles of SNPs 11-14 compared with the basal *LOXL1* promoter activity in human trabecular meshwork cells (hTMC), nonpigmented ciliary epithelial cells (hNPEC), optic nerve head astrocytes (hONHA), and HEK293T cells. Results are expressed as the ratio of Firefly luciferase to Renilla luciferase; the transcriptional activity of the empty pGL4.10 vector was set at 100% (Data represent mean values ± SD of 5 independent experiments; *p<0.05; unpaired two-tailed Student's t-test).

Supplementary Figure 6

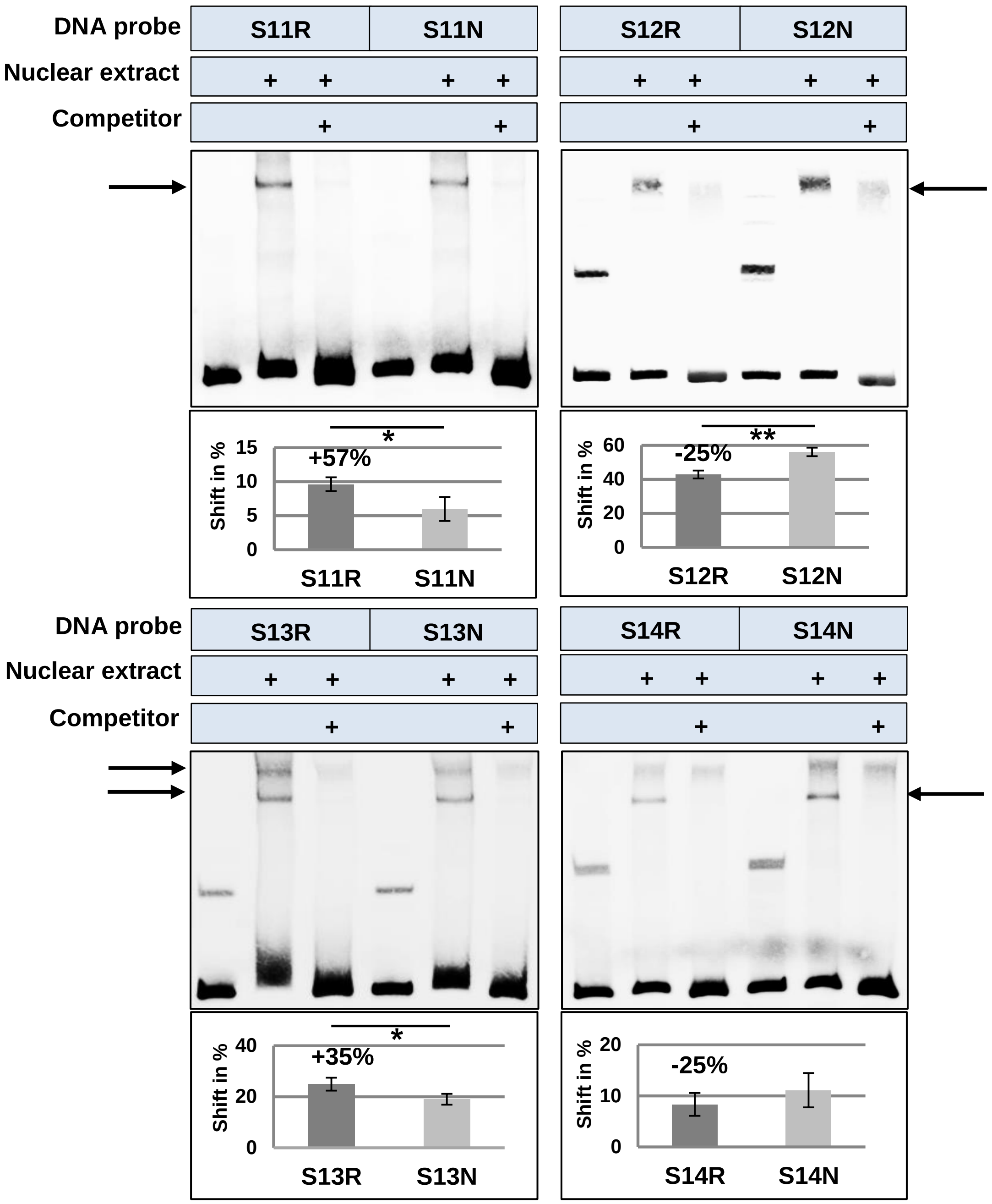

DNA fragments containing the risk (R) alleles of rs12905253 (S11), rs11638944 (S12), rs12441130 (S13), and rs11631579 (S14) show differential DNA-protein binding compared with fragments containing the non-risk (N) alleles. Electrophoretic mobility shift assays were performed with biotinylated DNA probes and nuclear extracts from human trabecular meshwork cells (hTMC) without and with a 200-fold excess competition with unlabeled DNA fragments. Arrows indicate specific DNA-protein complexes. Quantitative analyses of the shifted bands relative to the unshifted bands show mean values ± SD of 5 independent experiments (*p<0.05; **p<0.005; unpaired two-tailed Student‘s t-test).

Supplementary Figure 7

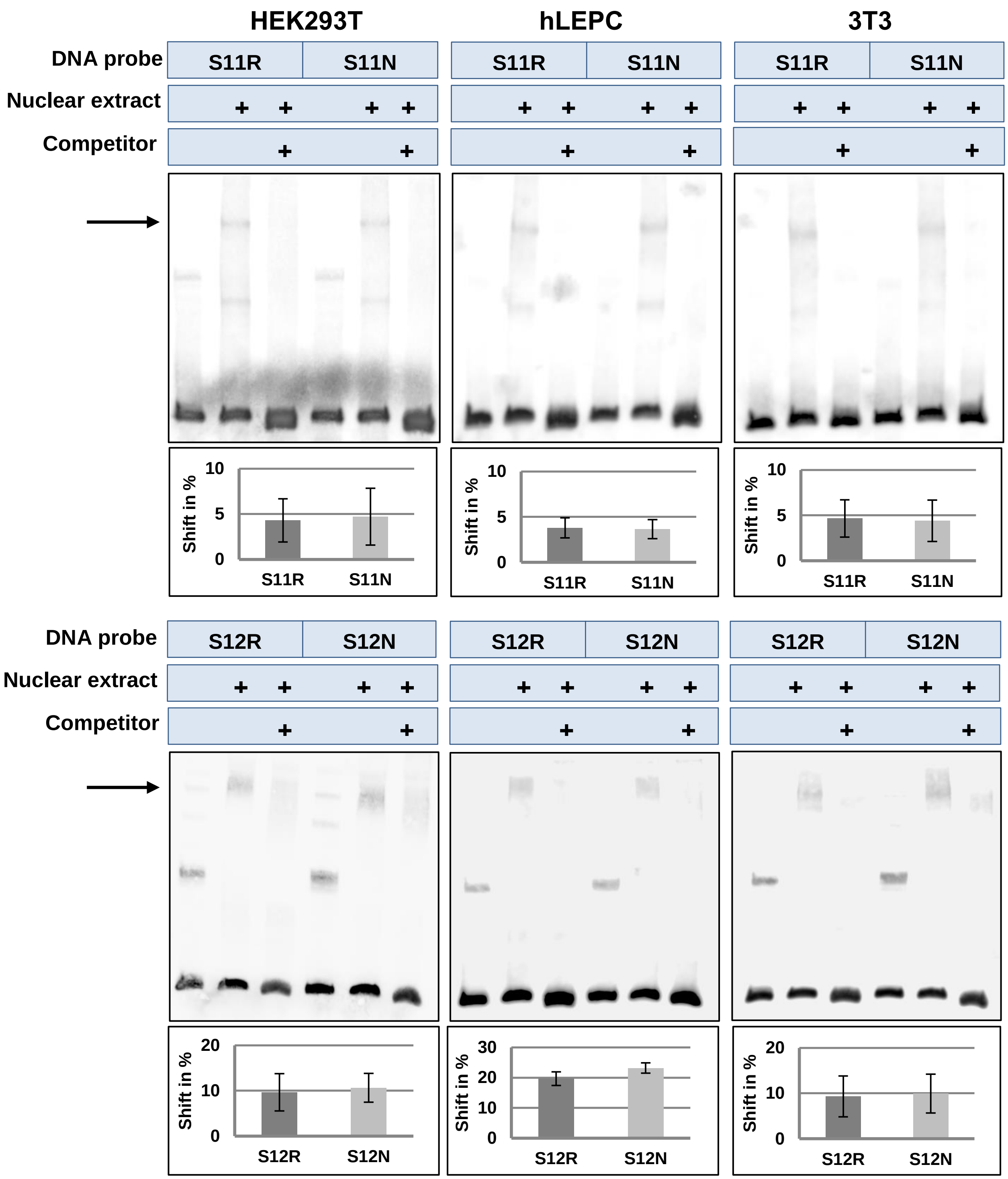

EMSA were performed with biotinylated DNA probes and nuclear extracts from HEK293T cells, human limbal epithelial cells (hLEPC), and 3T3 fibroblasts without and with a 200-fold excess competition with unlabeled DNA fragments. DNA probes containing the risk (R) and non-risk (N) alleles of rs12905253 (S11R) and rs11638944 (S12R) showed weak binding to nuclear proteins (arrows) without any allele-specific differences in DNA-protein binding efficiency. Quantitative analyses of the shifted bands relative to the unshifted bands show mean values ± SD of 3 independent experiments.

Supplementary Figure 8

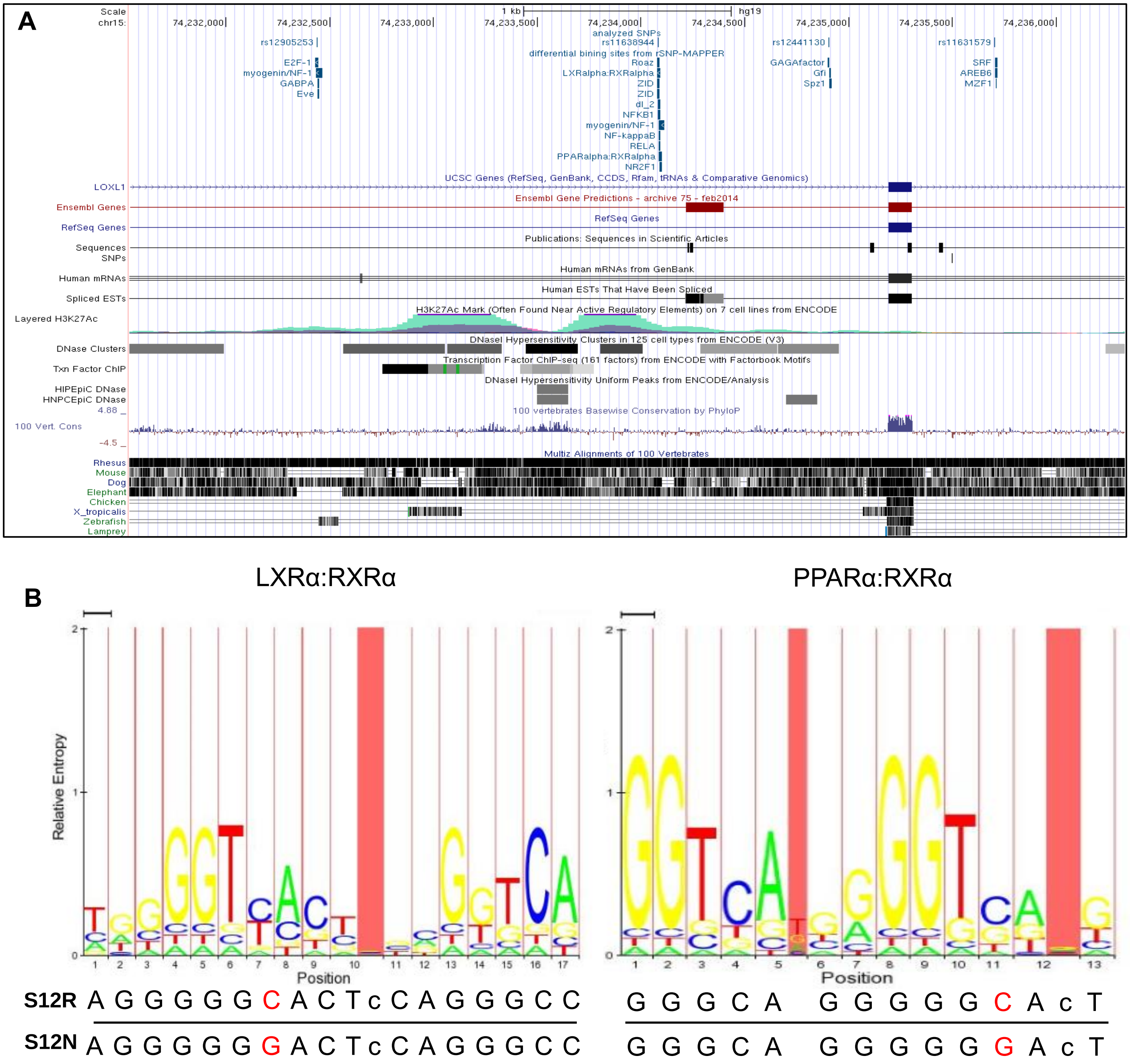

A. Regulatory elements located in the genomic regions of *LOXL1* (intron 1, exon 2, and start of intron 2) including SNPs 12905252, rs11638944, rs12441130 and rs11631579 and the alternative *LOXL1* transcript (Ensembl Genes) are depicted using a modified screenshot from the UCSC genome browser (http://genome.ucsc.edu). Shown are transcription factor binding sites as predicted by rSNP-MAPPER software overlapping SNP regions and ENCODE database, layered histone marks for H3K27Ac for 7 common cell lines (often found near regulatory elements), DNAse I hypersensitivity sites (indicating regions of open chromatin), and evolutionary conserved elements for 7 vertebrate species. **B**. Pictograms of transcription factor binding motifs and respective construct sequence variants for rs11638944 (S12N/R). Position weight matrix (PWM) of core half-site motifs were predicted by the rSNP-MAPPER software (Riva 2012).

Supplementary Figure 9

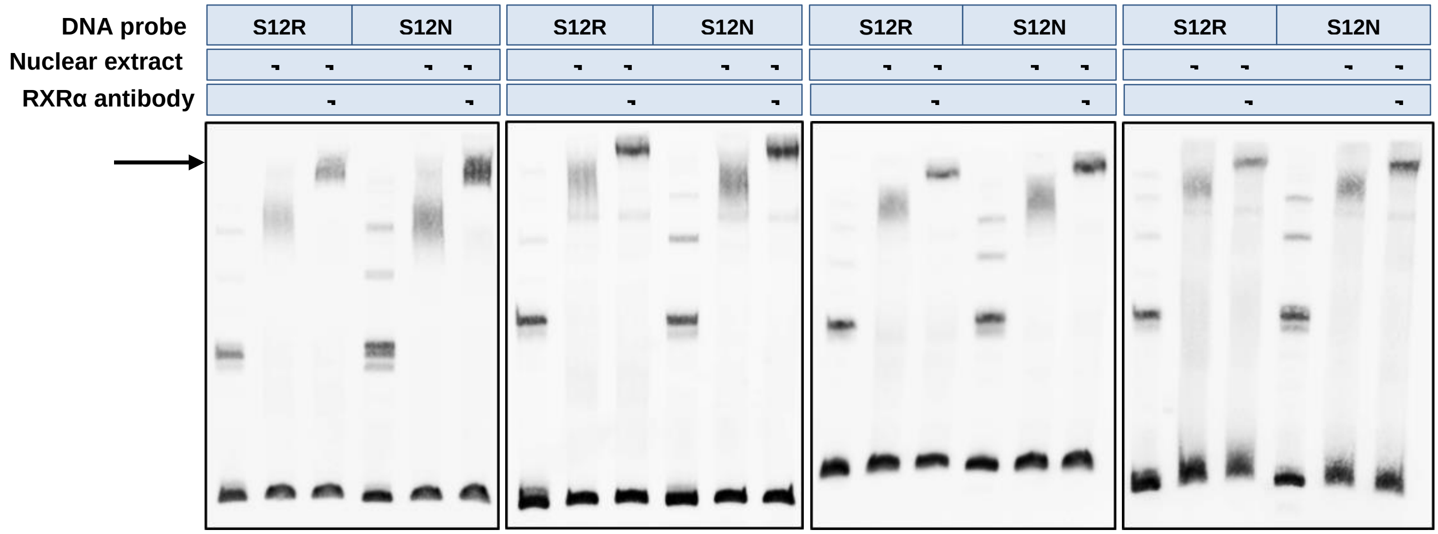

Independent supershift assays with biotinylated DNA probes and nuclear extracts from human trabecular meshwork cells. A specific antibody against RXRα disrupted the DNA-protein complexes to produce distinct supershifted bands (arrow) in a differential manner between DNA fragments containing the risk (R) alleles and fragments containing the non-risk (N) alleles of rs11638944 (SNP12).

Supplementary Figure 10

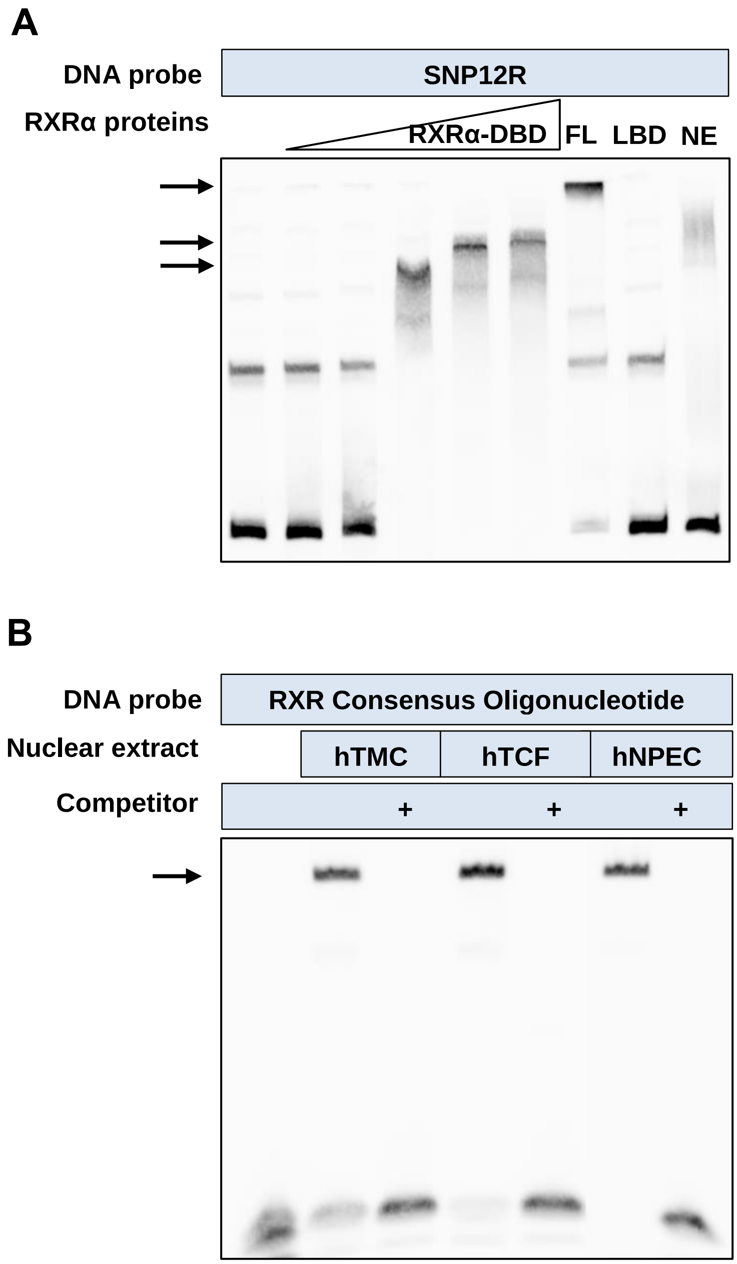

A. Control EMSA using SNP12R probe with increasing amounts of recombinant human RXRα-DNA binding domain (RXRα-DBD), human RXRα-ligand binding domain (LBD), human full-length RXRα (FL), and nuclear extract (NE) from human trabecular meshwork cells (hTMC) producing specific shifted bands (arrows) with RXRα-DBD and FL. **B**. Control EMSA using RXR consensus oligonucleotides and nuclear extracts from hTMC, human Tenon‘s capsule fibroblasts (hTCF), and human nonpigmented ciliary epithelial cells (hNPEC) producing specific shifted bands (arrow), which were completely inhibited with unlabeled DNA fragments (competitor).

Supplementary Figure 11

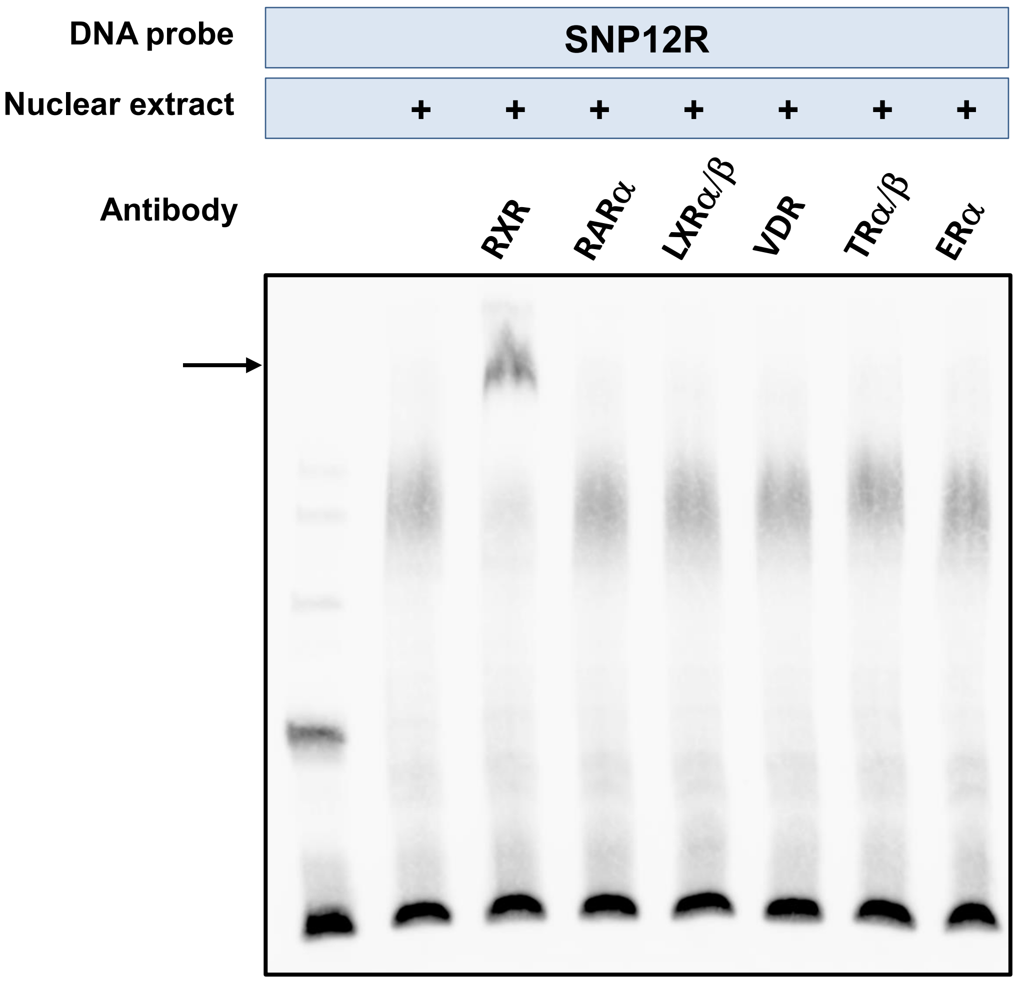

Supershift assay with biotinylated DNA probe containing the risk sequence of rs11638944 (SNP12R), nuclear extracts from human trabecular meshwork cells, and specific antibodies against known heterodimeric partners of RXRα, i.e., retinoic acid receptor (RAR)α, liver X receptor (LXR)α/β, vitamin D receptor (VDR), thyroid hormon nuclear receptors (TR)α/β, and estrogen receptor (ER)α. Only the specific antibody against RXRα disrupted the DNA-protein complexes to produce a distinct supershifted band (arrow).

Supplementary Figure 12

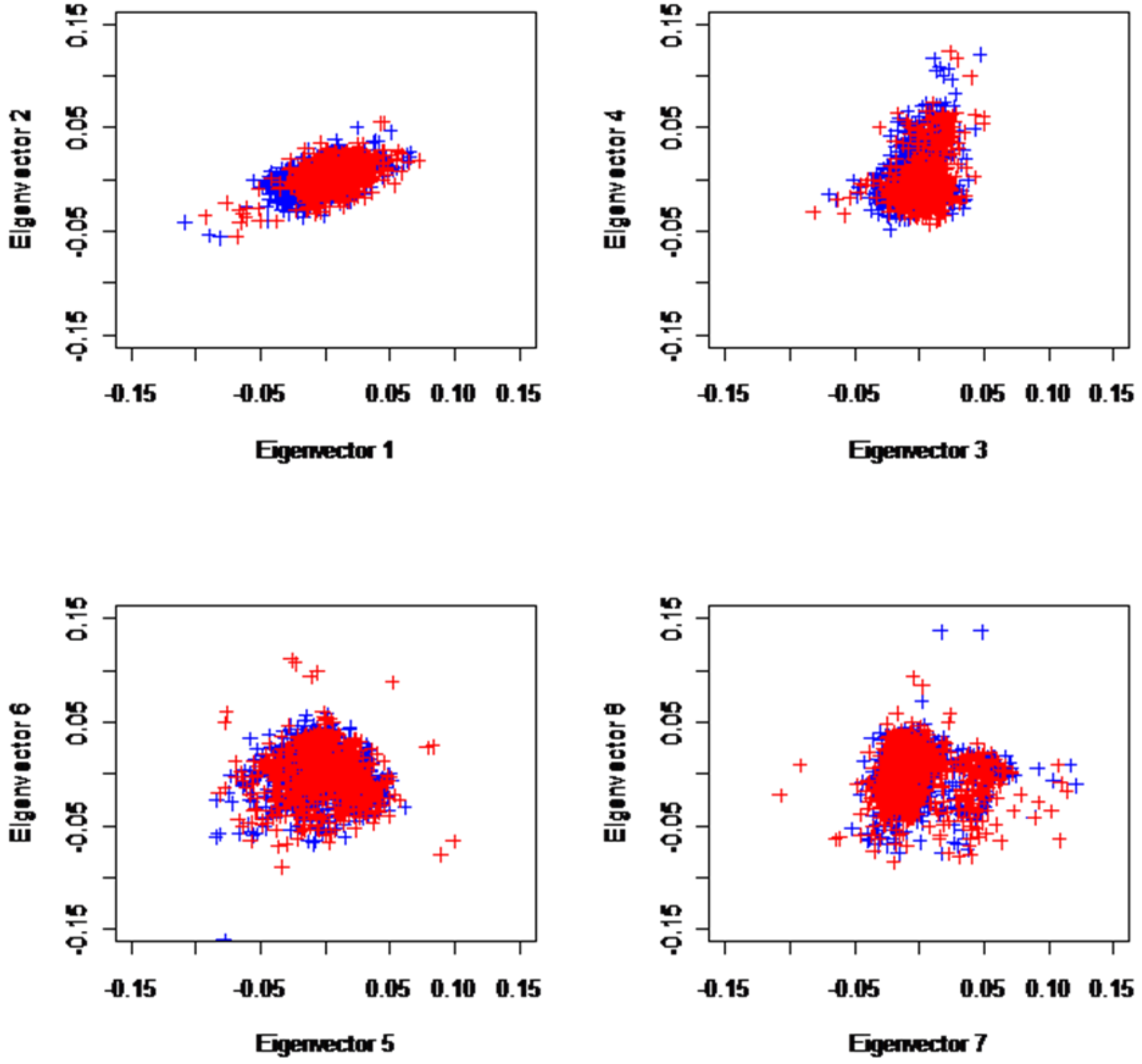

The first 8 principal components from the German data set as calculated by SMARTPCA (EIGENSOFT). 771 German PEX cases are shown in red, KORA German controls are in blue. German PEX cases and controls appeared to be well matched in terms of ancestry, as well as in overall assessment.

Supplementary Figure 13

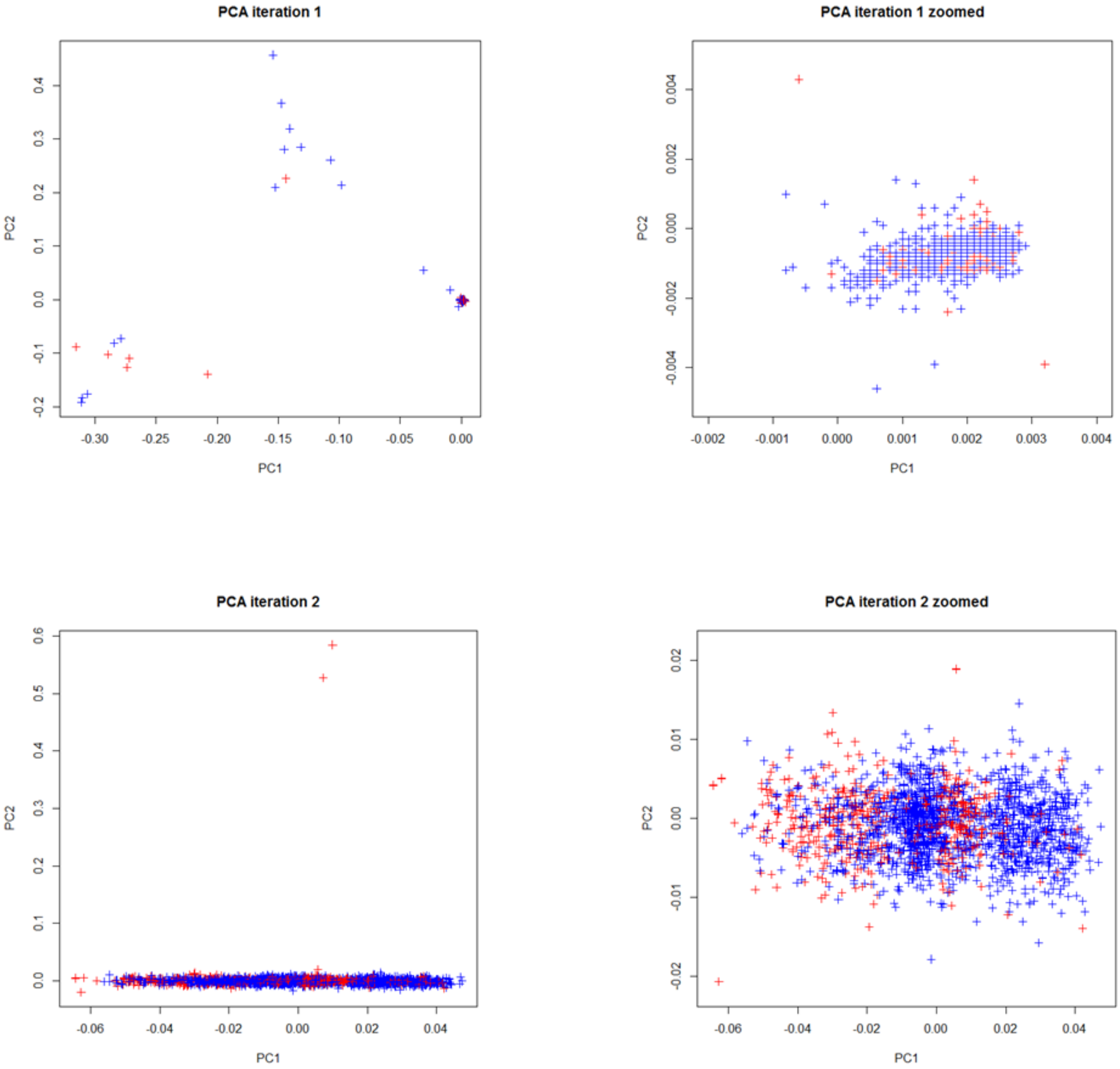

Primary Component Analysis (PCA) of Italian samples: The first 8 principal components from the Italian data set as calculated by SMARTPCA (EIGENSOFT). After the first PCA iteration, 21 samples with PC1 < 0.02 were removed as outliers. After the second iteration, the two case samples with PC2 > 0.5 were kept, as they were not expected to significantly inflate the results. In the second iteration, 473 Italian PEX cases are shown in red, 1545 Italian controls are shown in blue. The PCA shows well matched cases and controls in terms of ancestry as well as in overall assessment.

Supplementary Table 1. List of associated selected 14 SNPs with p-value<10^{-15} and the initial two coding SNPs, rs1048661 and rs3825942. Data of German cohort based on logistic regression model using gender and age as covariates. The columns are the original output from SNPTEST2.

rsid	chromosome	pos	allele A	allele B	average maximum posterior call	info	cohort 1 AA	cohort 1 AB	cohort 1 BB	cohort 1 NULL	all AA	all AB	all BB	all NULL	cases AA	cases AB	cases BB	controls AA	controls AB	controls BB	all maf	cases maf	controls maf	missing data proportion	het OR	het OR lower	het OR upper	hom OR	hom OR lower	hom OR upper	all OR	all OR lower	all OR upper	phenotype frequentist add score pvalue	phenotype frequentist add score info	phenotype frequentist add score beta 1	phenotype frequentist add score se 1
rs1048661	15	74219546	G	T	0.99704	0.99324	1142.4	834.65	157.91	0.025	1142.4	834.65	157.91	0.025	510.9	237.65	21.446	631.51	597.01	136.46	0.26943	0.18217	0.31866	0	0.49203	0.40678	0.59515	0.19426	0.12141	0.31081	0.47626	0.40894	0.55467	1.54E-09	0.99545	-0.8243	0.13648
rs3825942	15	74219582	G	A	0.99852	0.99338	1697.9	397.1	39.999	0.002	1697.9	397.1	39.999	0.002	714.16	50.822	5.012	983.74	346.27	34.987	0.11173	0.03951	0.15247	0	0.20217	0.14831	0.27558	0.19733	0.077001	0.50568	0.22866	0.17337	0.30158	2.81E-11	0.99414	-1.2573	0.18889
rs8023330	15	74220599	A	G	0.99438	0.99062	401.67	988.92	744.38	0.036	401.67	988.92	744.38	0.036	58.422	296.99	414.58	343.25	691.92	329.8	0.41974	0.26873	0.50493	0	2.5219	1.8516	3.4347	7.3856	5.4016	10.098	2.7754	2.424	3.1777	3.20E-18	0.98978	1.0477	0.12037
rs1550436	15	74221157	C	T	0.99603	0.99365	399.41	987.58	747.99	0.022	399.41	987.58	747.99	0.022	58.252	294.52	417.22	341.16	693.06	330.77	0.41837	0.2669	0.50381	0	2.4888	1.8263	3.3916	7.3875	5.4015	10.103	2.7889	2.4353	3.1938	2.09E-18	0.9936	1.0511	0.1201
rs2165241	15	74222202	T	C	1	1	797	980	358	0	797	980	358	0	447	278	45	350	702	313	0.39719	0.23896	0.48645	0	0.31008	0.2546	0.37764	0.11257	0.079936	0.15853	0.33149	0.28844	0.38096	1.22E-21	1	-1.1633	0.12173
rs28588430	15	74223430	C	G	0.99901	0.99816	798.16	977.27	359.57	0	798.16	977.27	359.57	0	447.2	277.57	45.229	350.96	699.7	314.34	0.39728	0.23898	0.48658	0	0.31133	0.25562	0.37918	0.11292	0.080245	0.1589	0.33134	0.28831	0.38079	1.30E-21	0.99943	-1.1618	0.12166
rs28617339	15	74223571	T	C	0.99936	0.99876	797.78	977.87	359.36	0	797.78	977.87	359.36	0	447.12	277.74	45.14	350.66	700.12	314.22	0.39732	0.23898	0.48665	0	0.31112	0.25545	0.37892	0.11267	0.080043	0.15859	0.33125	0.28823	0.38068	1.27E-21	0.99963	-1.1624	0.12169
rs4886778	15	74225388	C	A	0.99959	0.99933	841.99	950.99	342.02	0.004	841.99	950.99	342.02	0.004	466.99	261	42.005	375	689.98	300.01	0.38291	0.22404	0.47253	0	0.30376	0.24948	0.36985	0.11243	0.079202	0.1596	0.32228	0.27979	0.37124	8.25E-22	0.99964	-1.1675	0.12166
rs8027022	15	74226138	G	A	0.99911	0.99862	842.76	950.47	341.77	0.002	842.76	950.47	341.77	0.002	467.14	260.86	42.001	375.62	689.61	299.76	0.38267	0.22393	0.47221	0	0.30415	0.24981	0.37033	0.11266	0.079366	0.15993	0.32251	0.27998	0.3715	9.74E-22	0.99941	-1.1654	0.12165
rs2028386	15	74226708	C	G	0.99851	0.99761	843.13	951.19	340.68	0.005	843.13	951.19	340.68	0.005	467.26	260.86	41.879	375.87	690.33	298.8	0.38233	0.22378	0.47177	0	0.30397	0.24967	0.37009	0.11275	0.07939	0.16011	0.3228	0.28022	0.37184	7.62E-22	0.9977	-1.1706	0.12187
rs4337252	15	74226765	G	C	0.99953	0.99929	842.15	950.82	342.02	0	842.15	950.82	342.02	0	467	260.99	42.005	375.15	689.83	300.02	0.38287	0.22403	0.47248	0	0.30393	0.24962	0.37006	0.11247	0.079232	0.15966	0.32234	0.27983	0.3713	8.23E-22	0.99984	-1.1674	0.12164
rs12440667	15	74231439	C	T	0.99925	0.99913	395.04	988.55	751.4	0.001	395.04	988.55	751.4	0.001	57.001	295	418	338.04	693.55	333.41	0.41654	0.26559	0.5017	0	2.5226	1.8465	3.4461	7.435	5.4239	10.192	2.7841	2.4307	3.1887	1.55E-18	0.99831	1.0542	0.11999
rs12905253	15	74232437	G	A	0.99932	0.99911	394.98	988.43	751.59	0.002	394.98	988.43	751.59	0.002	56.961	295.04	418	338.02	693.39	333.59	0.41648	0.26556	0.50162	0	2.525	1.8482	3.4497	7.4357	5.424	10.193	2.7836	2.4304	3.1882	1.55E-18	0.99772	1.0545	0.12002
rs11638944	15	74234082	C	G	0.99932	0.99887	394.85	988.38	751.77	0	394.85	988.38	751.77	0	56.851	295.15	418	338	693.23	333.77	0.41641	0.26549	0.50155	0	2.5313	1.8524	3.459	7.4458	5.4303	10.209	2.7839	2.4305	3.1885	1.45E-18	0.99644	1.0559	0.12008
rs12441130	15	74234902	T	C	0.99924	0.99867	376.76	964.35	793.89	0	376.76	964.35	793.89	0	52.789	281.16	436.06	323.97	683.2	357.84	0.40231	0.25113	0.48759	0	2.5256	1.8296	3.4863	7.4785	5.4155	10.327	2.8377	2.4732	3.2558	8.01E-19	0.99547	1.0659	0.1203
rs11631579	15	74235704	A	G	0.99845	0.99764	393.54	988.86	752.6	0	393.54	988.86	752.6	0	56.227	295.76	418.01	337.31	693.1	334.59	0.41591	0.26508	0.501	0	2.5599	1.8711	3.5023	7.4948	5.4596	10.289	2.7836	2.4302	3.1884	1.41E-18	0.99568	1.057	0.12016

Data of Italian cohort based on logistic regression model using gender and age as covariates. The columns are the original output from SNPTEST2.

rsid	chrom	pos	allele A	allele B	average maximum posterior call	info	cohort 1 AA	cohort 1 AB	cohort 1 BB	cohort 1 NULL	all AA	all AB	all BB	all NULL	cases AA	cases AB	cases BB	controls AA	controls AB	controls BB	all maf	cases maf	controls maf	het OR	het OR lower	het OR upper	hom OR	hom OR lower	hom OR upper	all OR	all OR lower	all OR upper	pvalue	info	beta 1	se 1
rs8023330	15	74220599	A	G	0.98906	0.98272	440.62	935.97	549.39	0.023	440.62	935.97	549.39	0.023	19.122	170.18	231.69	421.49	765.79	317.7	0.47176	0.24754	0.53448	4.8985	3.0086	7.9754	16.075	9.8622	26.202	3.4901	2.9383	4.1456	3.75E-29	0.98195	1.1766	0.10499
rs1550436	15	74221157	C	T	0.99168	0.9869	439.2	933.94	552.84	0.022	439.2	933.94	552.84	0.022	19.067	168.9	233.03	420.13	765.05	319.8	0.4705	0.24588	0.53333	4.8644	2.9853	7.9264	16.056	9.8463	26.183	3.5052	2.95	4.1648	5.23E-29	0.98832	1.1706	0.10472
rs2165241	15	74222202	T	C	1	1	593	922	411	0	593	922	411	0	251	156	14	342	766	397	0.45275	0.21853	0.51827	0.27749	0.21893	0.35171	0.04805	0.02752	0.083895	0.25992	0.21745	0.31068	1.54E-31	1	-1.2172	0.10418
rs28588430	15	74223430	C	G	0.99912	0.99856	594.68	920.04	411.28	0	594.68	920.04	411.28	0	251.43	155.52	14.049	343.25	764.52	397.23	0.45239	0.21807	0.51793	0.2777	0.21909	0.35199	0.048283	0.027679	0.084224	0.25958	0.21714	0.31031	1.59E-31	0.99988	-1.2165	0.10415
rs28617339	15	74223571	T	C	0.99911	0.99856	594.72	920.05	411.23	0	594.72	920.05	411.23	0	251.47	155.48	14.047	343.24	764.57	397.18	0.45237	0.21802	0.51792	0.27756	0.21898	0.35182	0.048273	0.027672	0.084209	0.25951	0.21708	0.31024	1.58E-31	0.99988	-1.2166	0.10415
rs4886778	15	74225388	C	A	0.99961	0.99941	639.05	911.13	375.82	0	639.05	911.13	375.82	0	262.02	149.98	9	377.03	761.15	366.82	0.43167	0.1995	0.49661	0.28353	0.22401	0.35886	0.035304	0.017889	0.069673	0.25262	0.21027	0.30351	1.54E-32	0.99987	-1.2572	0.10585
rs8027022	15	74226138	G	A	0.9991	0.99859	639.8	910.57	375.63	0.001	639.8	910.57	375.63	0.001	262.07	149.93	9	377.73	760.64	366.63	0.43142	0.19944	0.49631	0.28411	0.22447	0.35958	0.035382	0.017928	0.069827	0.25283	0.21044	0.30377	2.13E-32	0.99868	-1.2548	0.10589
rs2028386	15	74226708	C	G	0.99586	0.99456	642.58	911.88	371.54	0.002	642.58	911.88	371.54	0.002	263.02	148.98	9	379.56	762.9	362.54	0.42964	0.19831	0.49435	0.2818	0.22263	0.35669	0.035824	0.018152	0.0707	0.25302	0.21053	0.3041	2.41E-32	0.9952	-1.2577	0.10622
rs4337252	15	74226765	G	C	0.9994	0.99901	639.04	911.2	375.76	0	639.04	911.2	375.76	0	262.1	149.9	9.002	376.94	761.3	366.75	0.43165	0.19941	0.49661	0.28318	0.22373	0.35842	0.0353	0.017888	0.069662	0.25248	0.21014	0.30334	1.38E-32	0.99932	-1.2585	0.10587
rs12440667	15	74231439	C	T	0.99364	0.99046	429.01	939.72	557.26	0.007	429.01	939.72	557.26	0.007	17.471	167.88	235.65	411.54	771.85	321.61	0.46671	0.24088	0.52988	5.1234	3.0857	8.5066	17.26	10.391	28.669	3.5521	2.9865	4.2247	1.29E-29	0.99092	1.1844	0.1048
rs12905253	15	74232437	G	A	0.99376	0.99047	429.1	939.66	557.23	0.009	429.1	939.66	557.23	0.009	17.471	167.87	235.66	411.63	771.79	321.58	0.46674	0.24087	0.52992	5.1246	3.0865	8.5087	17.266	10.395	28.678	3.5528	2.9871	4.2256	1.23E-29	0.99099	1.1844	0.10476
rs11638944	15	74234082	C	G	0.99387	0.99046	429.23	939.37	557.39	0.007	429.23	939.37	557.39	0.007	17.47	167.86	235.67	411.76	771.51	321.72	0.46673	0.24085	0.52992	5.128	3.0885	8.5144	17.266	10.395	28.679	3.5531	2.9874	4.226	1.16E-29	0.99128	1.1841	0.10469
rs12441130	15	74234902	T	C	0.99293	0.98927	394.98	926.89	604.12	0.012	394.98	926.89	604.12	0.012	12.472	160.88	247.64	382.51	766.01	356.47	0.44571	0.2207	0.50865	6.4415	3.5742	11.609	21.306	11.848	38.314	3.6554	3.0598	4.367	5.88E-31	0.9875	1.2284	0.10617
rs11631579	15	74235704	A	G	0.99337	0.98949	427.79	940.45	557.75	0.01	427.79	940.45	557.75	0.01	17.461	167.85	235.68	410.33	772.59	322.07	0.46626	0.24083	0.52932	5.1055	3.0745	8.4782	17.197	10.352	28.568	3.5452	2.9807	4.2166	1.20E-29	0.99131	1.1839	0.1047
rs3825942	15	74219582	G	A	1	1	1442	437	47	0	1442	437	47	0	418	3	0	1024	434	47	0.13785	0.00356	0.17542	0.016934	0.0054093	0.053011	-1	-1	-1	0.016808	0.005389	0.052426	9.30E-26	1	-1.7475	0.16654
rs1048661	15	74219546	G	T	0.99602	0.99158	973.47	789.23	163.29	0.012	973.47	789.23	163.29	0.012	265.48	148.4	7.124	707.99	640.84	156.16	0.28967	0.19316	0.31667	0.61755	0.49187	0.77534	0.12166	0.056666	0.26119	0.51662	0.42827	0.62319	2.75E-09	0.99223	-0.70867	0.11918

chrom=chromosome, pos=position based on UCSC Genome Browser (hg19), all=allele, maf=minor allele frequency, OR=odd ratio

Supplementary Table 2. Haplotype association analysis of the two coding SNPs rs1048661 and rs3825942 of the original publication (Thorleifsson et al. 2007) with the selected 14 SNPs in German (DE) and Italian (IT) cohorts. Genotypes of the two coding SNPs are in bold and underlined. A list of the 5 more frequent haplotypes in German and Italian cohorts is shown. The more frequent haplotypes are shown in lines 4, highlighted in red and correspond to the risk haplotypes (Association p-value=1,80-E-52 in German and P-value=2,62E-56 in Italian). Haplotype analysis was performed with PLINK (F_A=frequency affected; F_U=frequency unaffected; CHISQ=chisquare; P=p-value; OR=Odd Ratio; SE= standard error; L95-U95= 95% confident interval Lower and Upper value).

Nr.	Haplotype	Occurrence DE	Occurrence IT						
1	**TG**ACCGCAAGCCGCTA	1145	1171						
2	**GG**ACCGCCGCGCGCCA	59	83						
3	**GG**ACTCTCGCGCGCTA	86	46						
4	**GG**GTTCTCGCGTAGCG	2482	2188						
5	**GA**ACCGCAAGCCGCTA	475	556						
	Assoc (DE)								
	Haplotype	F_A	F_U	CHISQ	P	OR	SE	L95	U95
1	**TG**ACCGCAAGCCGCTA	0,1803	0,3176	94,68	2,24E-22	0,4726	0,07796	0,4056	0,5506
2	**GG**ACCGCCGCGCGCCA	0,01427	0,01355	0,03689	0,8477	1,053	0,2711	0,6192	1,792
3	**GG**ACTCTCGCGCGCTA	0,02659	0,01648	5,101	0,02391	1,63	0,2183	1,062	2,5
4	**GG**GTTCTCGCGTAGCG	0,7341	0,5055	232,4	1,80E-52	2,8225	0,0692	2,4643	3,2321
5	**GA**ACCGCAAGCCGCTA	0,04021	0,1513	123	1,38E-28	0,235	0,1402	0,1786	0,3093
	Assoc (IT)								
	Haplotype	F_A	F_U	CHISQ	P	OR	SE	L95	U95
1	**TG**ACCGCAAGCCGCTA	0,1921	0,3163	55,3	1,03E-13	0,5137	0,0906	0,4302	0,6136
2	**GG**ACCGCCGCGCGCCA	0,01775	0,02115	0,4271	0,5134	0,836	0,2745	0,4881	1,432
3	**GG**ACTCTCGCGCGCTA	0,01879	0,008974	6,331	0,01187	2,115	0,3044	1,164	3,84
4	**GG**GTTCTCGCGTAGCG	0,7589	0,5324	250	2,62E-56	3,5829	0,0836	3,0414	4,2212
5	**GA**ACCGCAAGCCGCTA	0,007307	0,176	177,1	2,13E-40	0,03447	0,3823	0,0163	0,07292
	Risk								
	Protective								

Supplementary Table 3. DNA primers used in this study.

3.1. DNA primers used for genotyping

Name	Sequence (5‘ - 3‘)	Product
rs1048661-3825942-F	CTTGCTCAACTCGGGCTCAGA	463bp
rs1048661-3825942-R	GGGCCGGTAGTACACGAAACC	
rs2165241-F	GCTCTGGTCCTTACCAGGTACTTGCAG	428bp
rs2165241-R	AATGTTTTTGACCCAAAATGAACTGTGG	
rs1550436-F	GTGGTATGCCGAGCCATATT	235bp
rs1550436-R	GGGAATGAGGCCAGTGAGGT	
rs8023330-F	CTCTGATCCTGGCTTTGGTG	304bp
rs8023330-R	CTCTAACCTCCTGCGCACTC	
rs28588430-F	CCTCGATGTGACCACTCCTG	231bp
rs28588430-R	CTGCCTGTTCCATGTTCCTT	
rs28617339-F	CTCCCTGGAGTTTCAGCTTG	356bp
rs28617339-R	GGTCAGACTGCAGGGGTTTA	
rs4886778-F	CTTAGAAAGCTGTGTCGGATCA	315bp
rs4886778-R	GGGAATTAAATGAGAAAAATAA	
rs8027022-F	AGCTGGGAACACATGGAAGA	261bp
rs8027022-R	TGATCATGAGTCCCGACAAA	
rs4337252-2028386-F	CAGTGCCACCAGACGTTTTA	277bp
rs4337252-2028386-R	GGTGAGTGGTATTATCTTTT	
rs12440667-F	TCACCAGGTCCAGGATCTTC	344bp
rs12440667-R	TTTCCAGGAAGGAACAATGG	
rs12905253-F	GGCAGGACATGGAAAACACT	292bp
rs12905253-R	CTCTTGTTGCGGGAAGTCTC	
rs11638944-F	GAATTAGAGGCCCCAGAACC	228bp
rs11638944-R	GCAGGAGTCTGAGCAGGAGT	
rs12441130-F	CCAGGTCTTTGTTCATGCTGT	252bp
rs12441130-R	CACCCCAAACATCCTCTCATA	
rs11631579-F	CAGGAAACTGAGGAGCAATGA	282bp
rs11631579-R	CCCTCTTTCCAGTGCAACATA	

3.2. DNA primers used for real-time PCR

Name	Acc. No.	Product	T_{an}	$MgCl_2$	Sequence (5‘ - 3‘)
LOXL1-F	NM_005576	112 bp	62°C	3.5mM	ACTACGATGTGCGGGTGCTACTG
LOXL1-R					TGGCTGAACTCGTCCATGCTGTG
LOXL1_AS1-F	NR_040066.1	166 bp	62°C	3.0mM	ACCCCAAAAGTCTGCTCTCAAG
LOXL1_AS1-R					ACAGAAAGAGCAAGGGACCAAG
LOXL1_E1A-F	NM_005576.3	181 bp	59°C	3.0mM	CGTGTACCGGCCCAAC
LOXL1_E1A-R					CCCTTGTTCCCCAGGATGT
RXRα-F	NM_002957.5	178 bp	62°C	3.0mM	GAGTTAGTCGCAGACATGGACAC
RXRα-R					TCAGGGTGCTGATGGGAGAATG
GAPDH-F	NM_002046.5	194 bp	64°C	3.0mM	AAGGTCGGAGTCAACGGATTTGG
GAPDH-R					ATGACAAGCTTCCCGTTCTCAGC
SNP12-F	NM_005576	148 bp	60°C	3.0mM	CCCAGAACCACTCCCCTTTA
SNP12-R					ATTGGAATGAAGAGGGAATATCTC
SNP13-F	NM_005576	121 bp	60°C	3.0mM	TTCATGCTGTTTTCCCTGCC
SNP13-R					GTGTGGGAGCTGCTAAGACT

3.3. DNA primers used for PCR

Name	Sequence (5‘ - 3‘)	Product
LOXL1_Pr-F	CAGCCCaagcttGGCCAGAAGAGCAG	1636 bp
LOXL1_Pr-R	TCAGGGTGCTGATGGGAGAATG	
LOXL1_E1-E2-F	CGTGGGCAGCGTGTAC	331 bp
LOXL1_E1-E2-R	CTGGCCAGACACTTCTCCTC	
Amplification for EMSA studies:		
S11RN-F	GGCAGGACATGGAAAACACT	292 bp
S11RN-R	CTCTTGTTGCGGGAAGTCTC	
S12RN-F	GAATTAGAGGCCCCAGAACC	228 bp
S12RN-R	GCAGGAGTCTGAGCAGGAGT	
S13RN-F	AGTAAGGACTCGAGGGAGTGC	232 bp
S13RN-R	AGGGAAGTCAGGCAGCAAGAG	
S14RN-F	CAGGAAACTGAGGAGCAATGA	282 bp
S14RN-R	CCCTCTTTCCAGTGCAACATA	

Acc.No, accession number; T_{an}, annealing temperature; GAPDH, glyceraldehyde-3-phosphate dehydrogenase; LOXL1_AS1, LOXL1 antisense RNA 1; LOXL1_E1A, LOXL1 transcript including exon 1A; LOXL1_E1-E2, LOXL1 region exon 1 to exon 2; LOXL1_Pr, LOXL1 Promoter; RXRα, retinoic X receptor alpha; aagctt: HindIII restriction site.

For preparation of biotinylated fragments for electrophoretic mobility shift assays (EMSA) primers were used 5'- labelled with biotin, while unmodified primers were used for the amplification of competitor fragments.

Supplementary Table 4. Predictions by rSNP-MAPPER (Riva 2012) of transcription factor (TF) binding sites potentially affected by rs12905253, rs11638944, rs12441130, and rs11631579. For each SNP, the Table shows the predicted binding scores for the sequences containing reference (non-risk) alleles and those containing the risk alleles as well as the score change; the identifier (as in the internal database of the tool) of the TF matrix model associated with the examined TF; the corresponding factor; the strand as well as start and end positions of the predicted TF binding site.

	Score			Model	Factor	Strand	Start	End
	reference allele	risk allele	change					
rs12905253	-	2.6	2.6	M00629	Eve	-	30	39
rs12905253	-	0.9	0.9	M00056	myogenin / NF-1	-	23	51
rs12905253	0.7	-	0.7	MA0062	GABPA	+	29	38
rs12905253	0.3	-	0.3	M00938	E2F-1	-	19	34
rs11638944	-	3.9	3.9	M00647	LXRalpha:RXRalpha	-	72	89
rs11638944	-	3.6	3.6	M00056	myogenin / NF-1	-	78	106
rs11638944	-	2.7	2.7	M00467	Roaz	-	71	84
rs11638944	3.4	0.8	2.6	M00085	ZID	-	72	83
rs11638944	-	2.6	2.6	MA0017	NR2F1	+	82	95
rs11638944	3.6	1.2	2.4	T01468	ZID	-	72	83
rs11638944	2.1	-	2.1	MA0023	dl_2	-	77	86
rs11638944	-	1.6	1.6	MA0061	NF-kappaB	-	78	87
rs11638944	1.5	-	1.5	MA0107	RELA	-	78	87
rs11638944	3.1	2	1.1	MA0105	NFKB1	-	77	87
rs11638944	4.2	5	0.8	M00518	PPARalpha:RXRalpha	-	80	93
rs12441130	4	-	4	M00723	GAGA factor	-	124	134
rs12441130	2	-	2	MA0038	Gfi	-	131	139
rs12441130	0.8	-	0.8	MA0111	Spz1	-	132	142
rs11631579	1	-	1	MA0083	SRF	-	179	190
rs11631579	-	0.7	0.7	M00083	MZF1	+	184	189
rs11631579	0.3	-	0.3	M00412	AREB6	-	181	191
